# Central giant cell granuloma of the mandibular condyle: A rare case and a literature review^☆^

**DOI:** 10.1016/j.heliyon.2019.e03085

**Published:** 2019-12-28

**Authors:** Gabriele Bocchialini, Luana Salvagni, Agostino Guerini, Andrea Castellani

**Affiliations:** a Maxillo-Facial Surgery Unit, Asst Spedali Civili, Brescia, Italy; b Department of Molecular and Translational Medicine, Section of Pathology, University Spedali Civili Di Brescia, Spedali Civili di Brescia, Unità Operativa di Anatomia Patologica, Brescia, Italy

**Keywords:** Dentistry, Surgery, Dental surgery, Bone, Oral medicine, Central giant cell granuloma, Condyle, Mandible, Intraosseous

## Abstract

**Introduction:**

Central giant cell granuloma is a benign intraosseous lesion; tumours in the condylar region are rarely reported.

**Case presentation:**

We present the case of a 60-year-old woman with preauricular swelling, limitation of joint motion and pain on only the right side.

**Discussion:**

The patient was evaluated based on her preoperative clinical manifestations, by orthopantomography and computed tomography (CT). CT revealed a lesion on the right condylar head. Surgery was scheduled based on this imaging finding, histological findings from an incisional biopsy specimen, and the patient's indications and symptoms.

**Conclusion:**

Of all reported cases of central giantcell granuloma, only five (including this case) were located in the mandibular condyle.

## 1 Introduction

Central giant cell granuloma is a benign intraosseous lesion first described by Jaffe [1]. It features cellular fibrous tissue with multiple haemorrhagic foci, multinucleate cells and trabecular bone (World Health Organisation [WHO]) [2]. These lesions constitute about 7% of all benign jaw tumours [3] and are divided into aggressive and non-aggressive types [4, 5]. They very rarely affect the mandibular condyle; the literature contains only five such cases, including this one [6]. The origin of this lesion type remains unknown; the lesion may be reactive, a developmental anomaly or a benign neoplasm [7, 8, 9]. We here report a particularly rare central giant cell granuloma in the right mandibular condyle; we describe its histopathological, radiological, clinical and surgical features.

## 2 Case presentation

**Figure 1:**
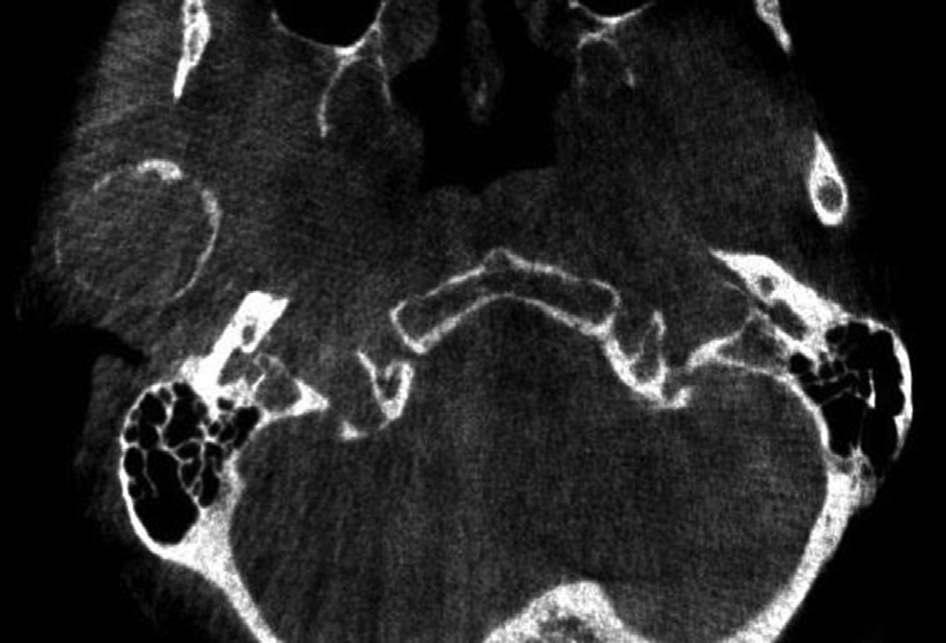
Preoperative Axial view of the lesion.

**Figure 2:**
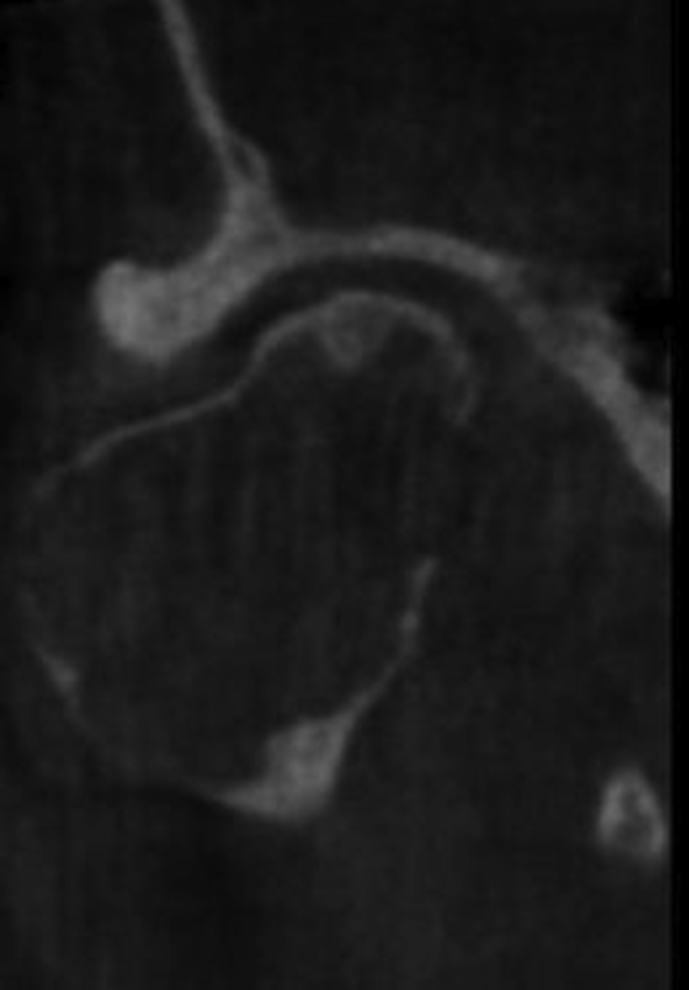
Preoperative Coronal view of the lesion.

**Figure 3:**
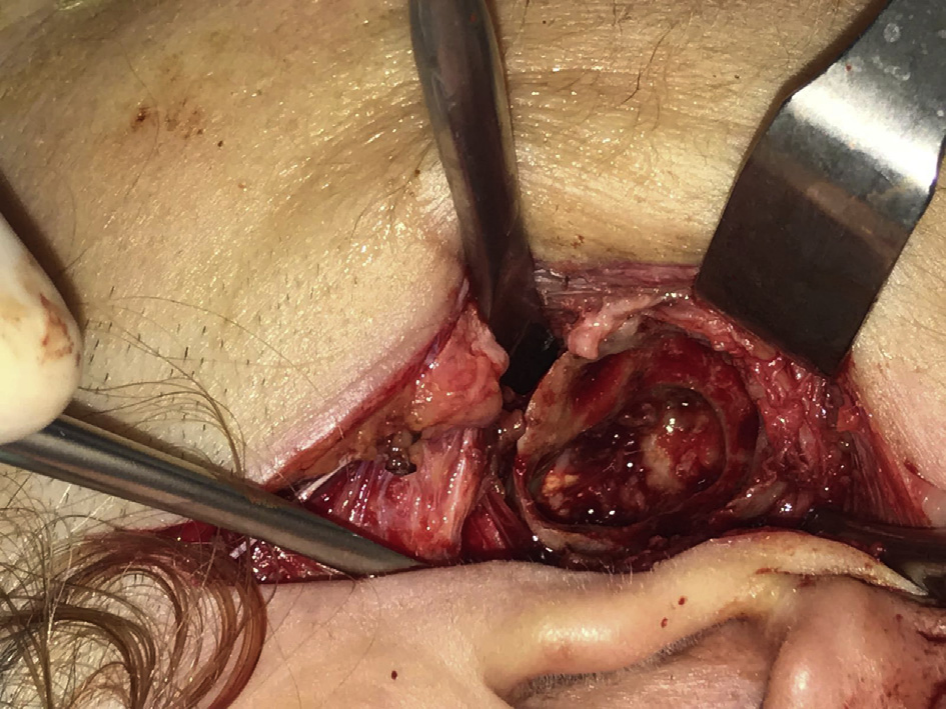
Intraoperative view of the preauricular approach of the temporomandibular joint (TMJ) region during removal of the lesion.

**Figure 4:**
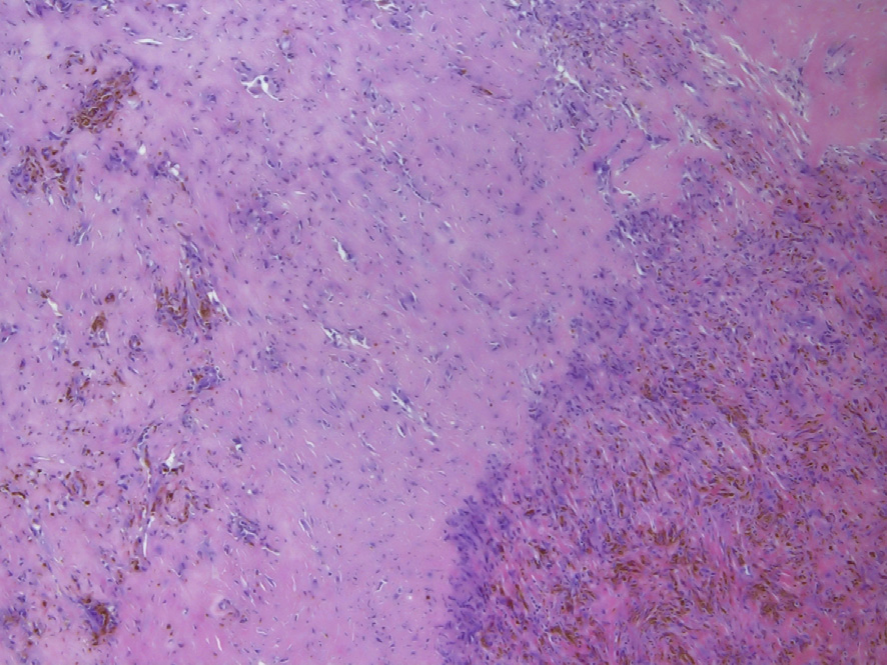
Fibroblastic proliferations with associated collagen, many blood vessels, and collections of epithelioid histiocytes that included numerous siderophages and multinucleate giant cells.

**Figure 5:**
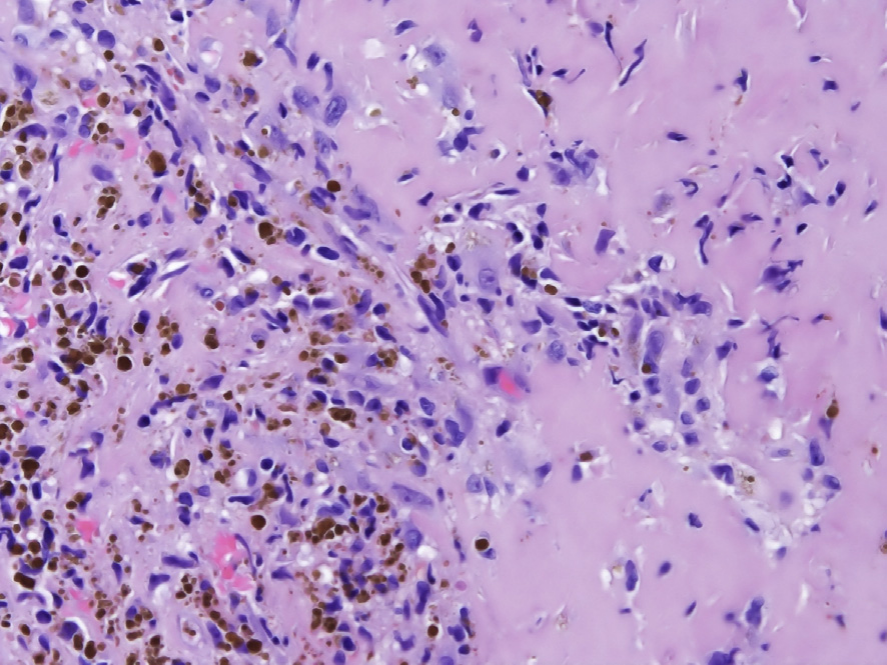
Fibroblastic proliferations with associated collagen, many blood vessels, and collections of epithelioid histiocytes that included numerous siderophages and multinucleate giant cells.

**Figure 6:**
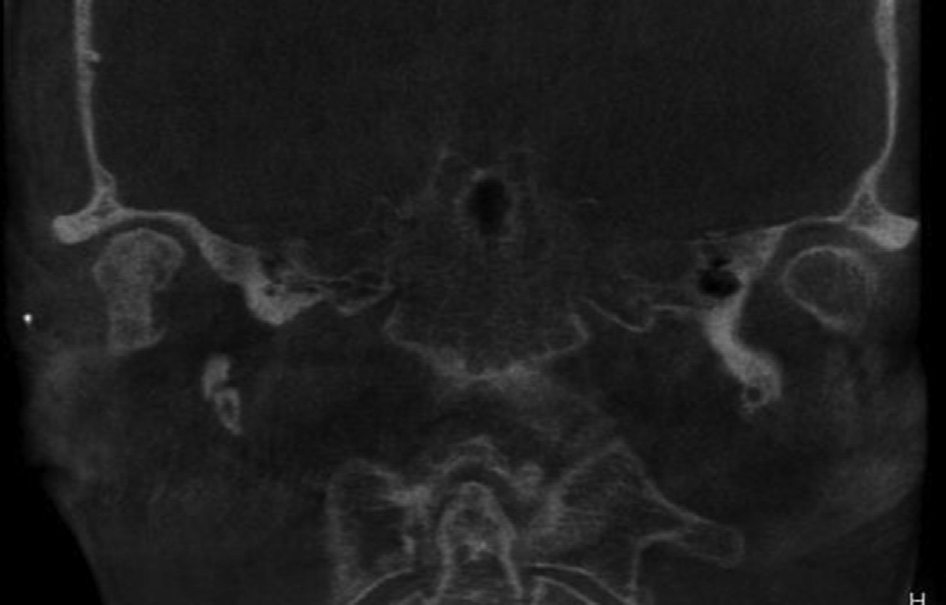
12 months follow up in axial view.

**Figure 7:**
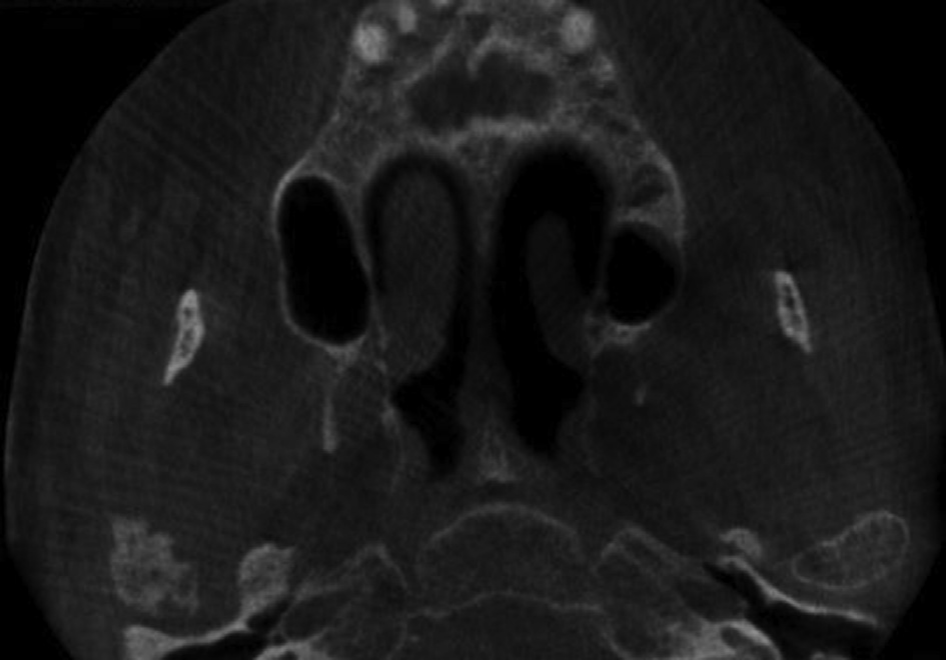
12 months follow up in coronal view.

A 60-year-old woman was referred to our institute with right-sided preauricular pain of 1-year duration that was aggravated by palpation, and a mouth-opening limitation. She experienced discomfort while chewing. The patient had no history of trauma or any other possibly relevant event prior to symptom onset. Physical examination did not detect facial nerve paralysis, or any hearing or facial sensation disturbance. Orthopantomography revealed distortion of the right condyle. Computed tomography (CT) revealed a large radiolucent lesion in the right condyle (Figures 1 and 2). The levels of parathyroid hormone, plasma phosphate, calcium and total protein were normal. An exploratory biopsy was performed with the patient under general anaesthesia. The histopathological diagnosis was central giant cell granuloma. Surgery was performed with the patient under general anaesthesia (after nasal intubation), using a right preauricular approach. Incision was followed by blunt dissection with preservation of the facial nerve. The lesion was completely removed (enucleated) and sent for histopathological evaluation(Figure 3). The surrounding soft tissues were intact. The specimen featured multiple fragments of 5 mL total volume, and contained woven bone, fibroblastic proliferations with associated collagen, many blood vessels, and collections of epithelioid histiocytes that included numerous siderophages and multinucleate giant cells. These histopathological findings allowed the diagnosis of central giant cell granuloma of the condyle (Figures 4 and 5). The patient has been followed for 12 months without recurrence (Figures 6 and 7).

Consent was gathered from patient investigated in this study.

## 3 Discussion

The WHO defines central giant cell granuloma as an intraosseous lesion featuring cellular fibrotic tissue with multiple haemorrhagic foci, multinucleated giant cells and trabecular bone [2]. Choung [4] and Ficarra [5] categorised such tumours as aggressive and non-aggressive. The non-aggressive form is more common, grows slowly, and usually presents clinically as painless swelling; only 20% of patients complain of pain or paresthaesia [5, 10]. Radiographically, such tumours present as well-defined unilocular or multilocular radiolucent lesions with undulating borders [7]. Aggressive tumours are encountered in younger patients and tend to grow faster; radiographically, they exhibit ill-defined borders and variable extents of cortical destruction [7, 8]. Of all central giant cell granulomas described in the literature, only five, including this case, were located in the mandibular condyle [6] (Table 1).

**Table 1:** Central giant cell granulomas described in the literature.

	Shensa, 1978	Tasanen, 1978	Abu-El-Naaj, 2002	Jadu, 2011
Age	15	59	15	31
Gender	M	M	F	M
Symptoms	Asymptomatic	Painless slow growing preauricular swelling	Painless swelling	Painful slow growing preauricular swelling
Imaging type	Panoramic, Laminagraphy	Laminagraphy	Panoramic, CT	Conventional, CT
Imaging features	Well Defined, Radiolucent, expansile	Multilocular tumor	Well defined, corticated, expansile	Well defined, expansile with a granular bone pattern
Management	Enucleation	Resection with costochondral reconstruction	Enucleation	Enucleation then resection after recurrence
Follow-up	N/A	21 Months	6 Months	4 Years

CT examination with a soft-tissue algorithm optimally determines the location, attachments, extensions and anatomical relationships of the lesion. The incidence of central giant cell granuloma in the general population is about 0.0001% [11]; 60% of cases present before the age of 30 years [8, 10]. Lesions in the mandibular condylar head are very rare [6]. Histological diagnosis is very difficult because the tumours are indistinguishable from brown tumours of hyperparathyroidism and giant cell lesions associated with genetic disorders such as cherubism, Noonan syndrome and neurofibromatosis 1[12,13]. Central giant cell granuloma may have a genetic etiology [13]. Its differential diagnosis includes tumours, cysts and tumour-like conditions of the preauricular area [8].

Treatments for condylar lesions range from curettage to lesion resection [6]; lesions in other locations respond to intralesional injection of corticosteroids or subcutaneous injection of human calcitonin [14, 15]. Recurrence rates range from 11% to 49% [16]. Our patient has been followed for 12 months without recurrence.

## 4 Conclusion

Central giant cell granulomas of the condyle are rare; this case is only the fifth reported in the English-language literature. For the previously reported cases (in one female and three males), the mean age at presentation was 30 years. Three cases were first treated via enucleation; one patient developed recurrence and underwent resection. One case was initially treated via resection and costochondral reconstruction [6]. Given the rarity of the condition, the reporting of all diagnosed cases with full case descriptions is essential.

## Declarations

### Author contribution statement

All authors listed have significantly contributed to the investigation, development and writing of this article.

### Funding statement

This research did not receive any specific grant from funding agencies in the public, commercial, or not-for-profit sectors.

### Competing interest statement

The authors declare no conflict of interest.

### Additional information

No additional information is available for this paper.
